# Analysis of NSAIDs in Rat Plasma Using 3D-Printed Sorbents by LC-MS/MS: An Approach to Pre-Clinical Pharmacokinetic Studies

**DOI:** 10.3390/pharmaceutics15030978

**Published:** 2023-03-18

**Authors:** Daya Raju Adye, Sachin B. Jorvekar, Upadhyayula Suryanarayana Murty, Subham Banerjee, Roshan M. Borkar

**Affiliations:** 1 Department of Pharmaceutical Analysis, National Institute of Pharmaceutical Education and Research, Guwahati 781101, India; 2 National Centre for Pharmacoengineering, National Institute of Pharmaceutical Education and Research, Guwahati 781101, India; 3 National Institute of Pharmaceutical Education and Research, Guwahati 781101, India; 4 Department of Pharmaceutics, National Institute of Pharmaceutical Education and Research, Guwahati 781101, India

**Keywords:** NSAIDs, 3D printed sorbent, fused filament fabrication, LC-MS/MS, pharmacokinetics

## Abstract

Analytical sample preparation techniques are essential for assessing chemicals in various biological matrices. The development of extraction techniques is a modern trend in the bioanalytical sciences. We fabricated customized filaments using hot-melt extrusion techniques followed by fused filament fabrication-mediated 3D printing technology to rapidly prototype sorbents that extract non-steroidal anti-inflammatory drugs from rat plasma for determining pharmacokinetic profiles. The filament was prototyped as a 3D-printed sorbent for extracting small molecules using Affinisol^TM^, polyvinyl alcohol, and triethyl citrate. The optimized extraction procedure and parameters influencing the sorbent extraction were systematically investigated by the validated LC-MS/MS method. Furthermore, a bioanalytical method was successfully implemented after oral administration to determine the pharmacokinetic profiles of indomethacin and acetaminophen in rat plasma. The *C_max_* was found to be 0.33 ± 0.04 µg/mL and 27.27 ± 9.9 µg/mL for indomethacin and acetaminophen, respectively, at the maximum time (*T_max_*) (h) of 0.5–1 h. The mean area under the curve (*AUC*_0*–t*_) for indomethacin was 0.93 ± 0.17 µg h/mL, and for acetaminophen was 32.33± 10.8 µg h/mL. Owing to their newly customizable size and shape, 3D-printed sorbents have opened new opportunities for extracting small molecules from biological matrices in preclinical studies.

## 1. Introduction

The fused filament fabrication (FFF) process involves printing various polymer materials into customized shapes layer-by-layer until a final structure is achieved [1]. FFF technology utilizes thermoplastic materials such as polylactic acid and polyvinyl alcohol, which can be used to fabricate customized delivery systems. FFF 3D printing is preferred due to its affordability and ability to print various thermoplastic polymeric filaments into tangible prototypes, including the ability to print personalized dosage forms and customized shapes. Recently, the extraction of small molecules from plasma samples using 3D printing technology has emerged, most notably in analytical chemistry [2]. Several analytical prototypes have been developed using 3D printing technology, including porous media columns [3], 3D-printed extraction sorbents for sample extraction from biological matrices [4], cooling interfaces for ultraviolet-light-emitting diode-based optical detectors [5], sensors [6], and microfluidic devices [7]. Therefore, the application of 3D printing technology in pharmaceutical analysis is of paramount interest in parallel with pharmaceutical research.

NSAIDs have been a major success in the pharmaceutical industry since their discovery, currently comprising 5% of all prescribed medications and ranking among the most commonly used over-the-counter drugs [8]. Despite the diversity within classes, their functions are relatively similar. NSAIDs are widely utilized for the management of pain, inflammatory conditions, postoperative surgical conditions, menstrual cramps, and also as antipyretics and analgesics [9,10]. During the early stages of the pandemic, NSAIDs were widely used in both inpatient and outpatient settings to alleviate symptoms of COVID-19, such as fever, body aches, and headaches [11]. As a result, some of these drugs have been included in the World Health Organization’s (WHO) list of essential medicines. Studies conducted in different populations have found no association between NSAID use, hospital admission, and adverse outcomes for COVID-19 patients [12,13,14]. Nevertheless, the use of NSAIDs has been linked to higher risks of complications, such as myocardial infarction, pleural empyema, and prolonged hospitalization, in patients suffering from non-COVID-19 respiratory infections [15,16,17,18]. The use of NSAIDs in older people is linked to a plethora of safety concerns, such as heightened risk of stroke, gastrointestinal bleeding, myocardial infarction, acute kidney injury, and bleeding disorders [15,16,17]. Therefore, it is crucial to employ appropriate sample extraction methods to detect and quantify low levels of NSAIDs in biological fluids. Most NSAID assays in biological fluids rely on liquid chromatography-mass spectrometry, ultra-high-performance supercritical fluid chromatography [19], gas chromatography-mass spectrometry, and high-performance liquid chromatography [20,21,22,23]. Prior to instrumental analysis, pretreatment is necessary, which involves selecting a suitable method for extracting specific NSAIDs from complex biological matrices. Classical sample preparation techniques such as liquid-liquid extraction (LLE) [24,25], solid phase extraction (SPE) [26,27], solid phase microextraction (SPME) [28], dispersive solid phase extraction (d-SPE) [29,30], and QuEChERS have been successfully used for the extraction of NSAIDs from biological matrices [31]. It is critical to appropriately prepare samples for analytical methods to ensure suitable selectivity and sensitivity. Analytical methods and the complexity of the sample matrix determine the number of steps required for sample preparation. In the past, sample preparation methods have been widely used to prepare samples from biological matrices, including protein precipitation and liquid-liquid extraction (LLE). Long processing times, many organic solvents, and interference from the sample matrix are the disadvantages of traditional sample preparation. Solid-phase extraction (SPE) is an alternative method for preparing samples to analyze compounds from different biological matrices, allowing for pure extracted samples, reproducible, rapid, high-throughput processes, and the reduced use of organic solvents. Modern sample preparation techniques, such as liquid-liquid and solid-phase microextraction and molecular imprinted polymers, are widely used to analyze complex matrices. The disadvantage of modern sample preparation techniques is that they are quite expensive, especially when analyzing many clinical samples. We designed a protocol for prototyping 3D-printed sorbents to prepare samples for bioanalysis that minimizes manual processing and reduces costs.

In the current study, the desired filament was fabricated using the hot-melt extrusion (HME) technique, followed by prototyping a 3D-printed sorbent via FFF-mediated 3D printing technology to extract and analyze NSAIDs samples using LC-MS/MS for pre-clinical pharmacokinetic studies in rodents. The objectives of this study were to: (a) create and exhibit a sample extraction technique using FFF-mediated 3D-printed sorbents produced from in-house extruded filaments, (b) assess the printed sorbent ability to analyze NSAIDs in rat plasma, and (c) enhance and verify a sensitive LC-MS/MS method for estimating the plasma concentrations of selected NSAIDs (indomethacin and acetaminophen) following oral administration in rats for pre-clinical pharmacokinetic evaluation. An innovative and affordable approach towards bioanalysis was developed by utilizing melt-extruded filament-derived customizable 3D-printed sorbents to extract selected NSAIDs from rat plasma to evaluate the pharmacokinetic parameters.

## 2. Experimental

### 2.1. Chemicals and Materials

Acetaminophen (99.5%), diclofenac sodium (99.7%) and indomethacin (99.9%) were purchased from Sigma-Aldrich (St. Louis, MO, USA). Affinisol^TM^ (hydroxypropyl methylcellulose HME 15 LV) was obtained from Du Pont Nutrition and Biosciences (Wilmington, DE, USA) as a gift sample. Triethyl citrate (TEC) and polyvinyl alcohol (PVA) were purchased from Sigma-Aldrich (St. Louis, MO, USA). LC-MS/MS-grade acetonitrile, methanol, and formic acid were obtained from J T Baker (New Jersey, United States). A Milli-Q filtration system (Millipore, Bedford, MA, USA) was used to obtain ultrapure water for analysis.

### 2.2. Extrusion of Affinisol^TM^ and PVA Blended Filaments through HME

Accurate weights of Affinisol^TM^ and PVA were mixed in a 1:1 (*w*/*w*) ratio with TEC (10% *w*/*w*) and then passed through a co-rotating twin-screw hot-melt extruder (HAAKE MiniLab II Thermo Scientific, UK) at 80 rpm and 165 °C. TEC was added as a plasticizer to preserve the flexibility of the extruded filament for FFF potential. The diameter of the extruded filaments was monitored after every 8.0 cm of the extruded length and maintained at an acceptable diameter of 2.74 (±0.11) mm for FFF-mediated 3D printing purposes, consistent with the print-core nozzle’s diameter of 2.85 mm.

### 2.3. Design & Prototyping of 3D-Printed Sorbents Using FFF

The prototypes of the 3D sorbents were designed using SOLIDWORKS software (SOLIDWORKS-2019, Dassault Systèmes SolidWorks Corporation, Waltham, MA, USA) in computer-aided design (CAD) format. The virtual design was then exported as a stereolithography (.stl) file to create a physical prototype. The intended dimensions for the 3D sorbent CAD file were 15.0 mm in length, 5.0 mm in width, 1.5 mm for the internal cut radius (up to length), 1.0 mm in thickness, and 1.62 mm in the internal arch that cut through the thickness. The 3D-printed sorbent was designed as a cylindrical flake that could be easily inserted into an Eppendorf tube for efficient analysis. These intended dimensions were fetched in .stl file format and used by the 3D printer slicer software (Ultimaker Cura Version 4.2.1, Singapore) to print objects in G-code file format for the final shape.

### 2.4. Differential Scanning Calorimetry (DSC)

To investigate the thermostability of the Affinisol^TM^ powder and PVA, 3D-printed raw sorbents and activated sorbents were subjected to DSC analysis (DSC-3, Mettler Toledo, Switzerland). Before analysis, each sample was weighed precisely and hermetically packed in an aluminum pan. Thermograms were recorded by holding all the samples at 25 °C for 1 min, followed by heating from 20 °C to 250 °C at a rate of 10 °C/min. All the samples were kept in equilibrium. Throughout the study, inert nitrogen was used as the purge gas at a flow rate of 20 mL/min.

### 2.5. Attenuated Total Reflectance-Fourier-Transform Infrared Spectroscopy (ATR-FTIR)

An ALPHA II Bruker spectrophotometer (Bruker, Billerica, MA, USA) using Opus software was used to perform the AT-FTIR analysis. Native Affinisol^TM^, native PVA, and 3D-printed sorbents before and after activation with water were analyzed by aligning the samples on the sample beam.

### 2.6. Brunner-Emmett Teller Analyser (BET)

The porosity and surface area of the raw and rinsed sorbents were analyzed using the Quantachrome Nova touch LX2 (Quantachrome Corp., Boynton Beach, FL, USA) through a Brunauer-Emmett-Teller (BET) analysis. The analysis was conducted with the aid of the Quantachrome TouchWin^TM^ version 1.22 software.

### 2.7. LC-MS/MS Analysis

Acetaminophen analysis was carried out on an Agilent 1290 Infinity II HPLC system coupled with an Agilent 6495C triple quadrupole mass spectrometer in positive ion mode (Agilent Technologies, Santa Clara, CA, USA). An Agilent jet stream electrospray ionization mode was used to ionize the ions in the positive mode. The source gas temperature was set to 290 °C; gas flow, 11 l/min; nebulizer, 45 psi; sheath gas temperature, 250 °C; sheath gas flow, 11 l/min; capillary voltage, +3500 V; and the nozzle voltage was set to 500 V. Instrument control and data acquisition were performed using a Mass Hunter workstation (version 10.1). A mass hunter workstation qualitative data application manager (Version 10.1) was used for qualitative data analysis. The chromatographic separation of acetaminophen and I.S. (indomethacin) was achieved on Zorbax Eclipse plus C18 (4.6 × 100 mm, 3.5 µm) held at 45 °C. The mobile phase was solvent A: 0.1% Formic acid in MilliQ water, and solvent B was 100% methanol. Separation was achieved using the gradient % B (time): 5% (0.00 min), 5% (0.20 min), 95% (5.00 min), 95% (8.00 min), 5% (10.00 min), 5% (12.00 min) with a flow rate of 0.5 mL/min. The injection volume was set to 10 µL and the autosampler temperature was maintained at 15 °C.

Indomethacin was analyzed using an Agilent 1290 Infinity II HPLC system (coupled with 6470 triple quadrupoles) equipped with an Agilent jet stream electrospray ionization source (Agilent Technologies, Santa Clara, CA, USA). The source gas temperature was set to 300 °C, the gas flow to 5 l/min, the nebulizer 45 psi, the sheath gas temperature to 250 °C, the sheath gas flow to 11 l/min, the capillary voltage +3500 V, and the nozzle voltage was set to +500 V. Data were acquired using the Mass Hunter Workstation software program (Version 10.1). A mass hunter workstation qualitative data application manager (Version 10.1) was used for qualitative data analysis. Chromatographic separation of indomethacin and I.S. (diclofenac) was achieved using a Zorbax Eclipse plus C18 (4.6 × 100 mm, 3.5 µm) (Agilent Technologies, Santa Clara, CA, USA) by maintaining a column temperature of 45 °C. The sample injection volume was set to 10 µL and the auto-sampler temperature was maintained at 15 °C during the analysis to minimize storage errors. A binary gradient mobile phase consisting of 10 mM ammonium acetate in water (A) and 100% methanol (B) followed the flow ratios of % B (time): 2% (0.00 min), 2% (0.20 min), 95% (5.00 min), 95% (8.00 min), 2% (10.00 min), 2% (12.00 min), respectively, with a flow rate of 0.5 mL/min. 

To detect the compounds, a positive mode of the AJS-ESI interface and multiple reaction monitoring (MRM) mode were utilized. The following Q1/Q3 transitions were monitored for the samples: *m*/*z* 358 > 110.9 for indomethacin, *m*/*z* 152 > 110 for acetaminophen, and *m*/*z* 296 > 215 for diclofenac sodium. 

### 2.8. Extraction Procedure

The 3D extraction sorbent developed in-house absorbs water and forms pores when dissolved. The activated sorbent was placed in a microcentrifuge tube, and the added drug samples were bound inside the porous structure. LC-MS/MS analysis was performed to determine the initial concentration of the mixture by examining the added concentrations. The sorbent was vigorously mixed by vortexing for 15 min after adding the drug solution. This process, known as the adsorption stage, allows the sorbent to absorb a drug solution. The mixture was in contact with the sorbent when the remaining solution was removed from the microcentrifuge tubes. To measure the amount of drug that was not absorbed, it was collected and centrifuged to remove unwanted particles from the residue. The supernatant was then subjected to LC-MS/MS for quantification. To prevent contamination of the unabsorbed mixture, the sorbent was transferred to a fresh tube. The sorbents were dried at room temperature and then placed in fresh microcentrifuge tubes. The desorption solvents were added to the sorbent after the adsorption procedure to release the adsorbed compounds. Vigorous mixing was assumed to release the adsorbed mixture into the desorption solvent during this stage. The desorption process required 15 min to release the adsorbed mixture from the sorbent, and the solvent was removed and transferred to a fresh tube for centrifugation. The concentration of the mixture that remained after desorption was evaluated by collecting and concentrating the supernatant and performing LC-MS/MS analysis.

### 2.9. In Vivo Assessment of 3D Sorbents

A 3D sorbent was used as the extraction device to evaluate the in vivo pharmacokinetics of acetaminophen and indomethacin. The Institutional Animal Ethics Committee of the National Institute of Pharmaceutical Education and Research, Guwahati (NIPER/CAF/IAEC/2022/60) approved all the animal experiments. For the pharmacokinetic study, the animal house was kept at a temperature of 25 ± 1 °C with a relative humidity of 50 ± 15%. The rats were maintained on a 12-h dark/light cycle and had free access to food and water. Prior to the study, the animals fasted for 12 h. To minimize variations in drug concentration in the plasma, 18 male Sprague-Dawley rats of similar weight and age were used. Rats were administered 50 mg/kg acetaminophen and 10 mg/kg indomethacin in saline through oral gavage to a different group. Blood samples were collected and thoroughly vortexed at 0.25, 0.50, 1, 2, 4, 6, and 8 h before being centrifuged at 5000 rpm for 20 min at 4 °C. The resulting plasma samples were separated and subjected to analysis using LC-MS/MS. The 3D extraction prototype was used to extract analytes from the plasma by introducing 90 µL of plasma spiked with 10 µL of an internal standard (I.S., 50 ng/mL), making up to 1 mL with deionized water, and adding the 3D extraction device for the adsorption process. After 15 min of adsorption in the vortexes, the remaining residue was collected for further analysis, the prototype was transferred into a fresh microcentrifuge tube, and desorption solvents (water and acetonitrile) were added in a 1:1 (*v/v*) ratio and vortexed for 15 min. Samples were collected after successful desorption and analyzed using LC-MS/MS.

#### 2.9.1. Standard Solutions and Plasma Samples for Extraction

Stock solutions of acetaminophen and indomethacin were prepared independently at 1 mg/mL. A stock solution (100 µL, 1 mg/mL) was added to 1 mL of water to generate a 1000 ng/mL concentration of each compound. A 1 mg/mL stock solution of diclofenac sodium (I.S.) in methanol was created and then diluted with deionized water to a total volume of 1 mL, resulting in a working concentration of 50 ng/mL.

#### 2.9.2. Calibration and Quality Control (Q.C.) Samples

To produce a calibration curve for acetaminophen (1–800 ng/mL) and indomethacin (1–500 ng/mL), primary aliquots of each compound (10 µL) were spiked into 90 µL of blank rat plasma containing 10 µL I.S. Additionally, the lower limit of quantification (LLOQ), low-quality control (LQC), medium-quality control (MQC), and high-quality control (HQC) samples were independently prepared at concentrations of 1, 3, 300, and 600 ng/mL for acetaminophen, and 1, 3, 250, and 400 ng/mL for indomethacin. All stock solutions were stored at 4 °C prior to use.

#### 2.9.3. LC-MS/MS Method Validation

The evaluation of compounds extracted from rat plasma was performed using a validated LC-MS/MS method that was developed per the U.S. Food and Drug Administration guidelines for validating bioanalytical methods [32]. The validation process included assessments of linearity, accuracy, precision, detection limit, quantification, and the carry-over effect. 

To assess the specificity and selectivity of the validated LC-MS/MS method and to determine the exclusion of endogenous interferences at the retention time of the compound and I.S., a total of six plasma samples and two plasma samples containing 50 ng/mL I.S. solution were subjected to extraction using the 3D sorbent. A calibration curve for rat plasma was constructed for standards over a range of 1–800 ng/mL for acetaminophen and 1–500 ng/mL for indomethacin. The peak area ratio of the drug and I.S. was plotted with a weighing factor of 1/*X* to assess acetaminophen linearity and linear regression for indomethacin. The minimum detectable concentration of analytes, also known as the lower limit of quantification (LLOQ), was established as the concentration at which the coefficient of variation (CV) is less than 20%, and the accuracy is within the range of 80–120%. Four different concentrations of quality control samples were used for six replicates to determine the intra- and inter-day precision and accuracy. As per the FDA guidelines, CV was limited to <20% for the LLOQ and ≤15% for the LQC, MQC, and HQC. Accuracy was calculated as the percentage difference between the observed and nominal concentrations of the QC samples. A comparison of the peak area ratios of analytes to I.S. of extracted samples (*n = 6*) and drug solutions of the same concentration was performed to evaluate the extraction recovery of molecules from rat plasma. LQC, MQC, and HQC concentrations were investigated for extraction recovery. The matrix effect of the LQC, MQC, and HQC samples was determined by deproteinizing the blank plasma samples and adding analytes and I.S. to the deproteinized sample. Furthermore, the same analyte concentrations were analyzed in aqueous solutions. The matrix factor was calculated using the following equation: (peak area of post-extracted standard analytes/peak area of the analyte in aqueous solution) × 100. To test the dilution integrity, the range of the standard compound concentrations was increased beyond the calibration range. Plasma samples with 1200 ng/mL of acetaminophen and indomethacin were individually prepared and then diluted two-fold and four-fold using blank plasma. The dilution integrity of concentrations was evaluated with %CV <15%. A series of injections was performed to assess the carry-over effect of acetaminophen and indomethacin. The injections included a blank solvent sample, a higher concentration standard sample, a blank plasma sample extracted with the 3D sorbent, and a higher concentration sample extracted with 3D sorbent from plasma. The thresholds for carry-over effects were 20% of the mean extracted LLOQ signal and 5% of the I.S. response. The stability of acetaminophen and indomethacin in rat plasma was evaluated at six low- and high-quality control sample replicates. Benchtop and post-operative stability studies were conducted at 8 h at ambient temperature and 15 °C for 24 h, respectively. Quality control samples were stored at −20 °C and thawed at room temperature every 24 h for freeze-thaw stability, and after three freeze-thaw cycles, samples were analyzed.

## 3. Results and Discussion

### 3.1. Extrusion of Affinisol^TM^ and PVA Blended Filaments through HME

Affinisol^TM^ and PVA have existed almost in the amorphous format, where the former follows the lowest to the highest processing temperature range between 135–190 ℃, and the latter at 200 °C, respectively. In addition, the glass transition temperature (Tg) of Affinisol^TM^ is reported to be 115 °C, yet it persists in its stable form up to its highest processing temperature range of 135–190 ℃. Accurate weights of Affinisol^TM^ and PVA were mixed in a 1:1 (*w*/*w*) ratio with TEC (10% *w*/*w*) to ensure filament stability within the processing temperature range without degradation. The mixture was then extruded through a co-rotating twin-screw hot-melt extruder (HAAKE MiniLab II Thermo Scientific, UK) at 80 rpm and 165 °C. TEC was added as a plasticizer to maintain the flexibility and rigidity of the fabricated filaments for FFF-mediated 3D printing. In addition, they are opaque and tolerant to the desired printability.

### 3.2. Design & Prototyping of 3D-Printed Sorbents Using FFF

**Figure 1:**
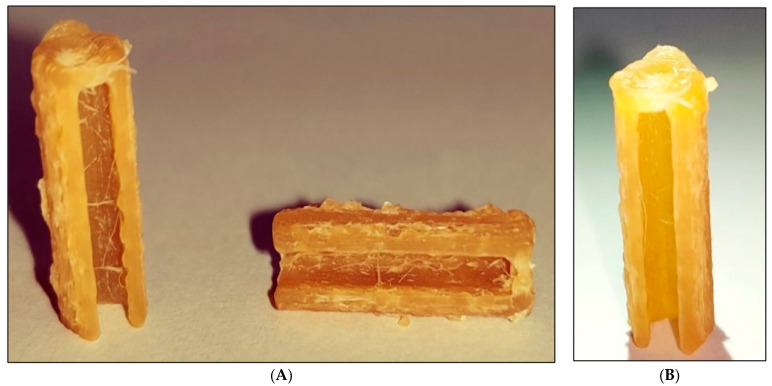
Fused filament fabricated 3D sorbent. (**A**) Raw sorbent before activation, vertical and horizontal view, and (**B**) rinsed sorbent after activation.

In-house, lab-manufactured cylindrical 3D-printed sorbents were produced, with a length of 15.0 mm and width of 5.0 mm and a specially designed internal cut radius from end-to-end up to the length of the sorbent. This specially designed internal cut radius prevents unusual sorbent breakage during the adsorption and desorption processes. The sorbent architecture was molded to occupy less space with a better adsorption profile, followed by recovery. Both were in a small volume of a microcentrifuge tube. The 3D sorbent architecture resembles a cylindrical shape, is pale yellowish in color, has a characteristic odor owing to the melting of polymers, is nonfragile, and has a rigid texture. Surface strandlines were visible at the sorbent surface, possibly due to the FFF mediated layer-by-layer printing. The 3D sorbents before activation with water and after activation for adsorption and desorption are shown in (Figure 1).

### 3.3. Characterizations of Sorbent and Selection of Extraction Method

The preparation of samples plays a crucial role in the analysis of compounds using high-end chromatographic techniques to identify chemicals in different matrices and determine their concentrations both qualitatively and quantitatively. In the past, sample preparation methods were limited by lengthy procedures, tediousness, extensive use of organic solvents, environmental hazards, etc. An ideal system is less expensive, requires a minimum volume/usage of organic solvents, and is quick and easy to use. In modern extraction techniques, minimal organic solvents must be used, the operating time should be reduced, and the extracted substances should be purified. Thus, the disadvantage of current sample preparation methods is that they are relatively expensive for analytical purposes for a significant portion of clinical samples. Thus, low-cost innovative methods with the adaptation of cutting-edge technologies for extracting analytes from biological matrices are the need of the hour. Various 3D-printed devices have been employed to aid in extracting analytes from biological matrices. These devices serve as sorbents, sorbent holders, membrane separators, phase separators, and extraction chambers [2,33,34].

**Figure 2:**
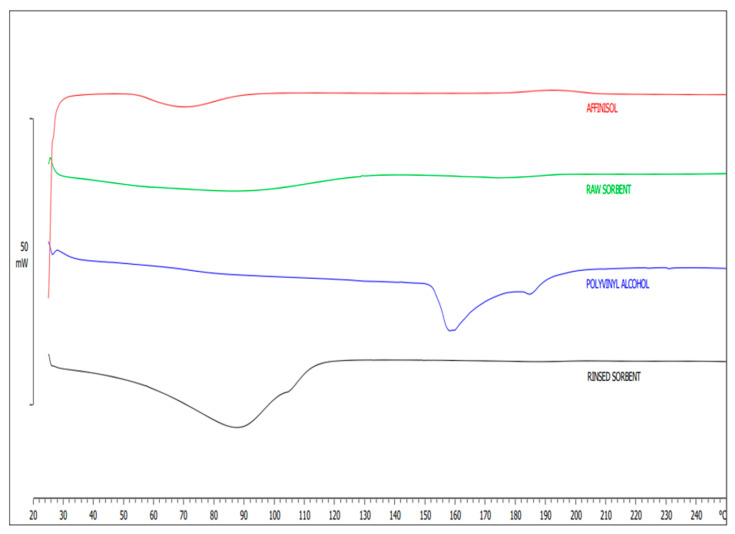
Over laid DSC thermograms of Affinisol^TM^ (HPMC HME 15 LV), raw sorbent, polyvinyl alcohol and rinsed sorbent.

Therefore, our other round of development of 3D-printed sorbents has enabled the fabrication of customized 3D sorbents for small-molecule extraction, particularly NSAIDs. In this study, we used the HME technique to fabricate a customized filament for 3D printing a sorbent for extracting NSAIDs, using PVA, Affinisol^TM^, and TEC as plasticizer. The sorbent was activated through immersion in water, which removed unreacted PVA, resulting in a flexible and porous material. The activated sorbent enables efficient adsorption and extraction of small molecules. The fabricated filaments were extruded using polymers Affinisol^TM^ and PVA to aid HME. DSC analyses were conducted to confirm the thermal stability, followed by ATR-FTIR studies to investigate the chemical interactions between the polymer functional groups. In DSC analysis, the printed 3D sorbent was found to be thermally stable, with no further thermal degradation observed in the 3D-printed extraction sorbent. Under raw and rinsed conditions, the fabricated sorbent could withstand a temperature range of 20–250 °C without altering its physicochemical properties, as evident from DSC analyses in Figure 2. In addition, a blunt peak was observed in the temperature range of 80–110 °C due to moisture evaporation, which was bound to the rinsed sorbent. Based on this observation, it can be concluded that both Affinisol^TM^ and polyvinyl alcohol polymers are stable throughout the thermal event, starting from HME extrusion to FFF-mediated 3D prototyping of the device.

**Figure 3:**
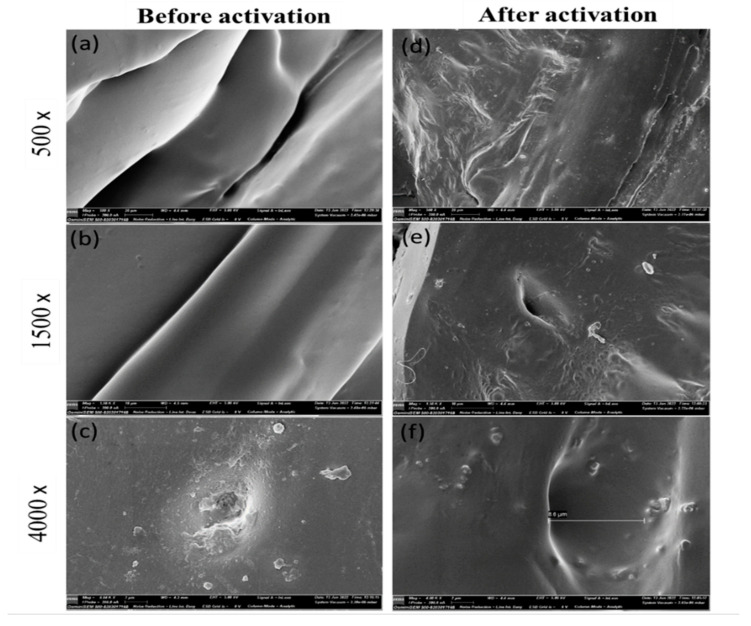
SEM image of 3D FFF printed sorbent. Before activation (**a**–**c**) and activated sorbent after rinsing with water (**d**–**f**).

The activation process using water resulted in the formation of a microporous structure on the surface of the sorbent by removing PVA [2,35]. Qualitative and quantitative DSC analyses were employed to measure the partial removal of PVA crystals from the extraction device. The DSC thermograms (Figure 2) show a mild endothermic peak at around 165 °C for PVA. However, this endothermic peak was not observed in the thermogram of the washed sorbent, indicating the removal of PVA from the extraction device during the activation process. This indicated that PVA was either completely or partially removed from the polymer composite [2], which involved interactions between the drug molecules and polymers and reversible adsorption on the surface of the extraction device. Prototyped 3D extraction devices formed pores after activation for 10 min with double-distilled water and sonication, as observed in Figure 3 after the sorbent activation. The sorbent desorbs molecules from the prototype by adding a solvent when activated. Affinisol^TM^, a modified form of HPMC [36], is known to exhibit excellent compression properties and adequate swelling behavior, can support high levels of drug loading, and is considered non-toxic [37]. The hydrogen bonds between the polymer chains are disrupted when radial water uptake is introduced to a highly viscous HPMC matrix that can swell, resulting in the physical adsorption process. Water entered the modified HPMC and sloughed into the hydrogen bonds between the polymer chains. The forces between the chains decreased as water continued to flow between them. The polymer swells because the chains initially gain rotational freedom and occupy additional spaces. The pores formed on the surface of the extraction device were confirmed by SEM before and after activation. Drug molecules are absorbed inside the voids of the modified HPMC polymer because of the penetrating water filling the spaces with them, and hydrogen bonds between the polymer chains and the molecules of the drug caused this to occur [37]. PVA was used to create a microporous structure on the surface of the sorbent, while TEC provided the necessary flexibility for filament extrusion and printing.

After adsorbing the drug molecules onto the surface of the extraction device and allowing for drying, acetonitrile and double distilled water were mixed in a 1:1 (*v/v*) ratio and vortexed for up to 15 min to recover the drug molecules from the extraction device. Water causes the device to swell, and organic solvents would effectively remove drug molecules. The extraction device before activation presented an extremely dense, rigid surface morphology under SEM (Figure 3a,c), while (Figure 3d–f) represents its rinsed version. However, after the PVA was removed, the same sorbent displayed a less dense, flappy network structure. Based on the minimal amount of PVA from the extraction device, Affinisol^TM^ functions as an activated cartridge for effective sorption and desorption. PVA crystals were visible, and a partial reduction in PVA crystals was observed on the activated sorbent (Figure 3d–f). The SEM images presented in Figure 3e,f depict the formation of surface pores on the sorbent after activation in water. Before and after activation, the printed layer of the composite was visible under low magnification. Following the release of PVA from the sorbent, the activated sorbent becomes porous, making the residual substance more flexible. A magnification of 4000× revealed the plane-printed layer surface on the raw sorbent.

**Figure 4:**
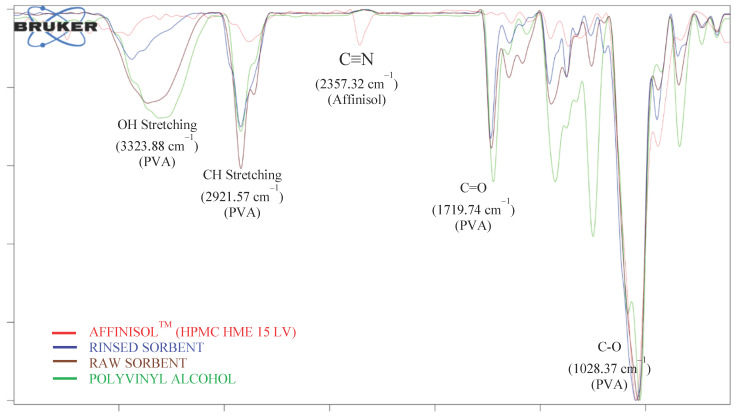
Overlaid ATR-FTIR spectra of Affinisol™ (HPMC HME 15 LV), rinsed sorbent, raw sorbent and polyvinyl alcohol.

ATR-FTIR analysis of the rinsed sorbents revealed a decrease in the distinctive absorbance bands of PVA OH stretching vibrations at 3323.88 cm^−1^, C-H at 2921.57 cm^−1^, C=O at 1719.74 cm^−1^, and C-O at 1028.37 cm^−1^ (Figure 4), which clearly illustrates that PVA was removed from the sorbents. Similar to this, after activation with water, the extraction devices showed characteristic absorbance bands of Affinisol^TM^ at 2357.32 cm^−1^ for C≡N, 2882.03 cm^−1^ for C-H, 1452.88 cm^−1^ for C-N, and 1046.55 cm^−1^ for C-O (Figure 4). This confirmed the material integrity following the successful fabrication of FFF-mediated printed extraction devices after extrusion through HME. After the extrusion process, Affinisol^TM^ retained its physicochemical properties based on ATR-FTIR analysis.

**Figure 5:**
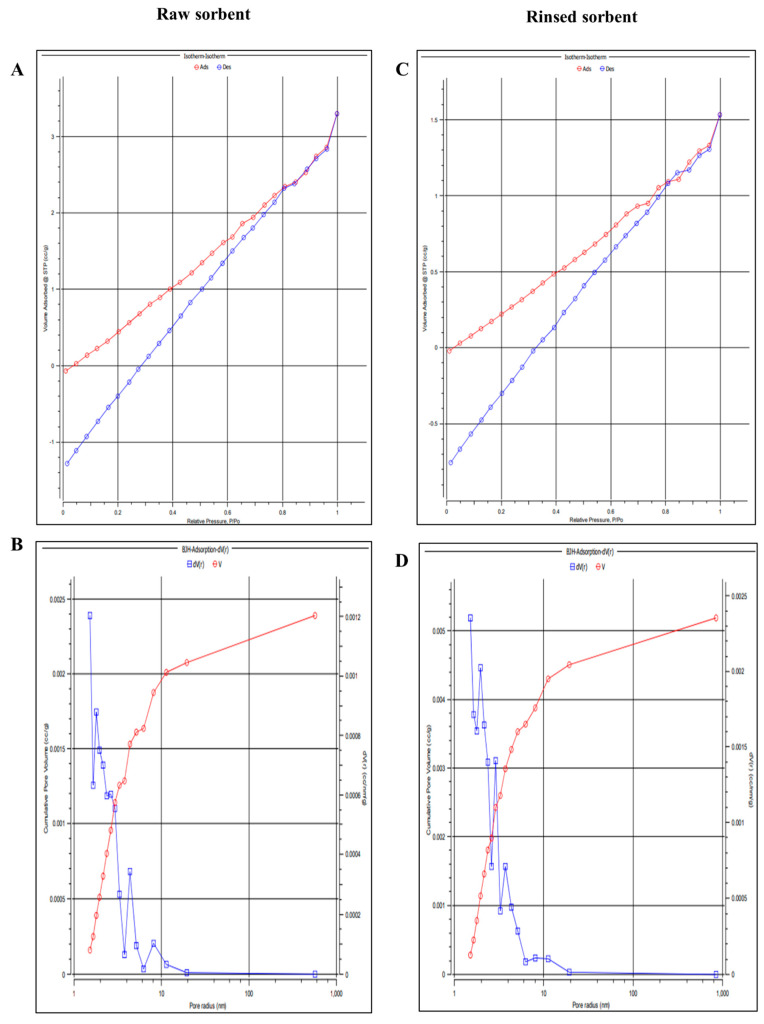
Characterization of the mesoporous structure of raw sorbent (**A**,**B**) and rinsed sorbent (**C**,**D**). BET isotherms of raw sorbent (**A**) and rinsed sorbent (**C**). Cumulative pore volume distribution of both (**B**) raw sorbent and (**D**) rinsed sorbent. The pore volume indicates the total volume of the pores at a specific diameter, in cc/g as evaluated from BET measurements.

The SEM images in Figure 3e,f demonstrate that activated sorbents have pores and grooves that enhance sorption. The porosity of the rinsed sorbent was quantitatively examined using a BET analyzer (Emmett–LX gas sorption analyzer) due to these characteristics. In addition, the adsorption/desorption isotherms were analyzed using the traditional helium void volume method for space measurements and nitrogen sorption, and the obtained data are displayed in Figure 5 for the raw and rinsed sorbents. Assessment of the mesoporous structure of raw sorbent (Figure 5A,B) and rinsed sorbent (Figure 5C,D), including BET isotherms of raw sorbent (Figure 5A), rinsed sorbent (Figure 5C) was evident through the profiling of cumulative pore volume distribution of both (Figure 5B) raw sorbent and (Figure 5D) rinsed sorbent. The microporous structure of the activated sorbent was subjected to adsorption by placing it in a microcentrifuge tube containing indomethacin and acetaminophen solutions in the rat plasma. To determine the unbound concentrations of indomethacin and acetaminophen, LC-MS/MS analysis was utilized. Following centrifugation, the sorbents were rinsed and dried using deionized water. Desorption of the solvent was carried out in fresh tubes using the sorbents. The resulting supernatant from the collected solvent was then centrifuged and introduced into the LC-MS/MS system to quantify the concentrations of indomethacin and acetaminophen. Subsequently, the 3D-printed sorbent extraction device was used to determine the plasma concentrations of indomethacin and acetaminophen after administering 50 mg/kg of acetaminophen and 10 mg/kg indomethacin to rats for pharmacokinetic profiling. 

Male Sprague-Dawley rats (*n = 6*) were used for pharmacokinetic studies, and oral doses of indomethacin and acetaminophen were separately administered to overnight-fasted rats. Blood was collected through retro-orbital puncture at different time points, and plasma was separated and stored for further analysis. Plasma containing indomethacin and acetaminophen was added to the microcentrifuge tube containing the activated extraction device for adsorption and vortexed for 15 min with 1 mL of water, after which the analytes present in the plasma were bound to the surface of the cartridge. After adsorption for 15 min, the remaining solvent was transferred into a fresh tube and centrifuged to precipitate the matrix. The resulting supernatant was collected and introduced into an LC-MS/MS system to quantify the unbound concentration. The sorbent was transferred into a fresh microcentrifuge tube for desorption using acetonitrile and water and vortexed for 15 min to complete the process. The solution was collected, followed by centrifugation, and the supernatant was filtered through a 0.2µm filter. The resulting filtrate was then subjected to LC-MS/MS analysis to measure the indomethacin and acetaminophen concentration in the plasma. 

### 3.4. Extraction Conditions

Various parameters that impact extraction efficiency, including sorption time and temperature, desorption solvent, and desorption time were investigated. For this assessment, activated sorbent was added to a microcentrifuge tube containing 100 ng/mL of indomethacin and acetaminophen and the effects were observed. Subsequently, the sorbents were transferred to a different tube for desorption, and the resulting solvent was centrifuged. The resulting supernatant was then subjected to LC-MS/MS analysis. 

#### 3.4.1. Adsorption and Desorption Time, Rpm, and Temperature

**Figure 6:**
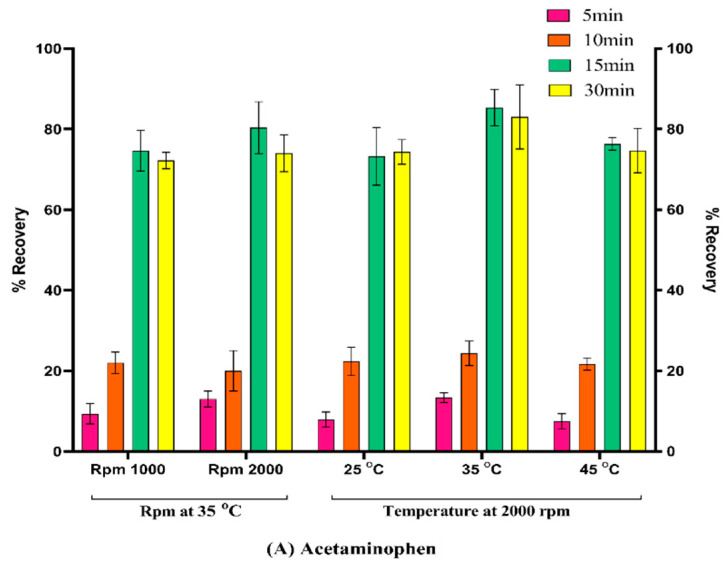

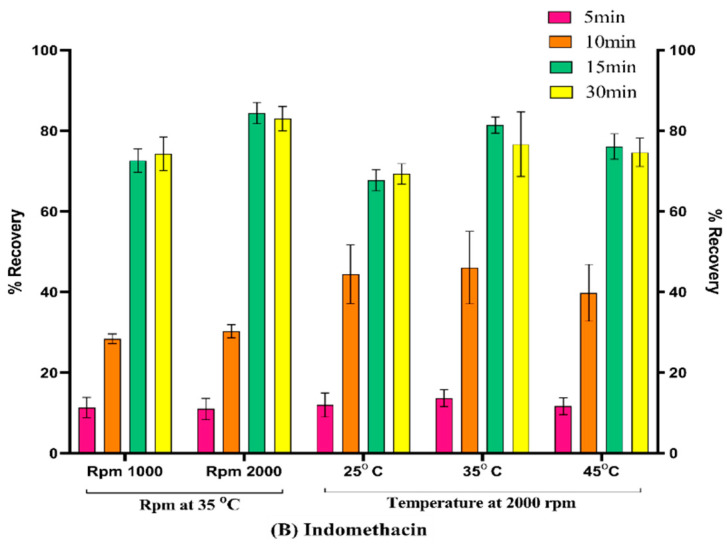
Optimization of extraction recovery of (**A**) acetaminophen and (**B**) indomethacin by using RPM and temperature.

To evaluate the sorption and desorption times, RPMs, and temperatures, activated sorbent was introduced into separate microcentrifuge tubes, each containing 100 ng/mL of indomethacin and acetaminophen in plasma. The main step to be optimized is the adsorption process, which can increase the adsorption efficiency of the sorbent after activation. The adsorption process was optimized at 1000 rpm and 2000 rpm at 25, 35, and 45 °C. To accomplish desorption, the sorbents were transferred to a fresh tube containing extraction solvent and subjected to incubation at 25, 35, and 45 °C, with corresponding rpm values of 1000 and 2000. To evaluate the recovery of indomethacin and acetaminophen, LC-MS/MS analysis was performed at different time intervals (5, 10, 15, and 30 min). Figure 6 illustrates that 35 °C, 2000 rpm, and 15 min were identified as the optimal incubation temperature, rpm, and time for both the sorption and desorption stages, resulting in the satisfactory recovery of acetaminophen and indomethacin. 

#### 3.4.2. Desorption Solvents

**Figure 7:**
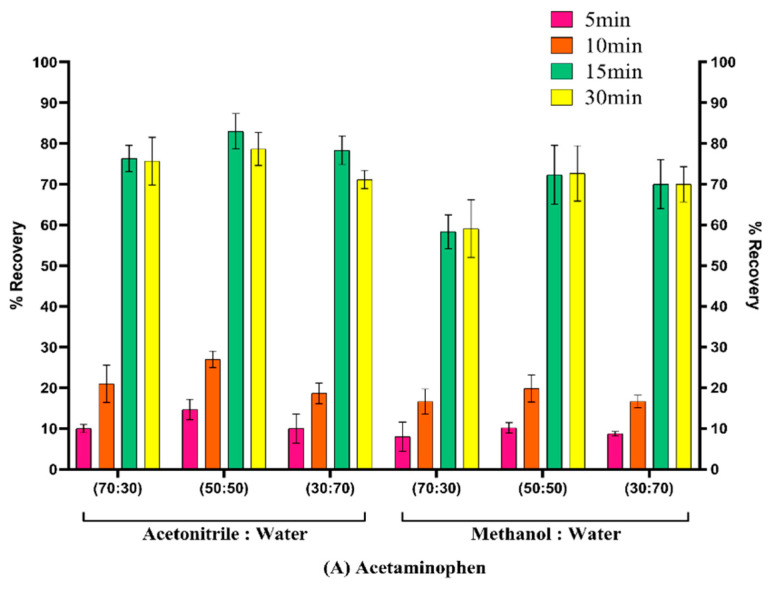

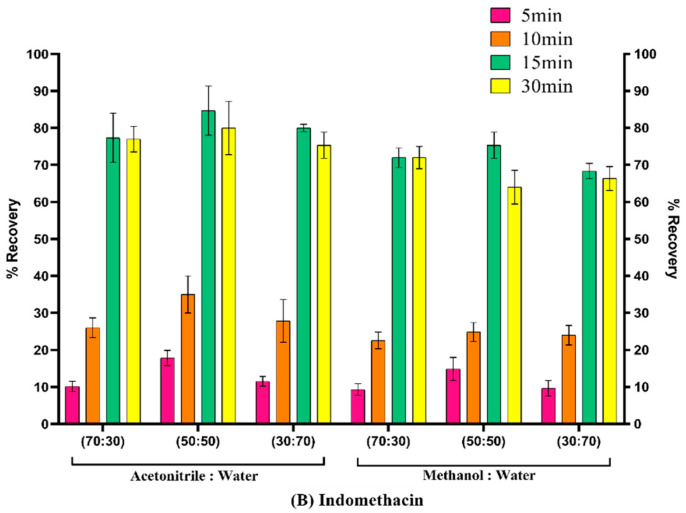
Optimization of extraction recovery of (**A**) acetaminophen and (**B**) indomethacin by using solvents acetonitrile: water (70:30, 50:50, 30:70) and methanol: water (70:30, 50:50, 30:70).

Effective drug recovery necessitates desorption, a critical stage in the extraction process. After sorption, an extraction solvent was used to extract indomethacin and acetaminophen from the sorbent. In this study, acetonitrile, methanol, and water were used as desorption solvents. Various water ratios were tested with acetonitrile, methanol, and water, and the ratio of water to acetonitrile (70:30, 50:50, and 30:70 *v/v*) led to extraction recovery rates exceeding 70% (Figure 7). In conclusion, desorption solvents containing 1:1 (*v/v*) acetonitrile and water provided satisfactory recoveries. The effect of the water content on analyte extraction was further investigated. As an organic solvent, acetonitrile was used to extract indomethacin and acetaminophen that had been embedded in the pores of the sorbent. Low percentages of indomethacin and acetaminophen were observed in the absence of water. The organic solvents used to extract indomethacin and acetaminophen from the activated sorbents did not affect the sorbent structure. The sorbent was tested for the removal of indomethacin and acetaminophen before activation. Nevertheless, LC-MS/MS analysis demonstrated that over 1% of the medication was bound to the raw sorbent.

### 3.5. LC-MS/MS Method Validation and Its Application to Pharmacokinetic 

**Figure 8:**
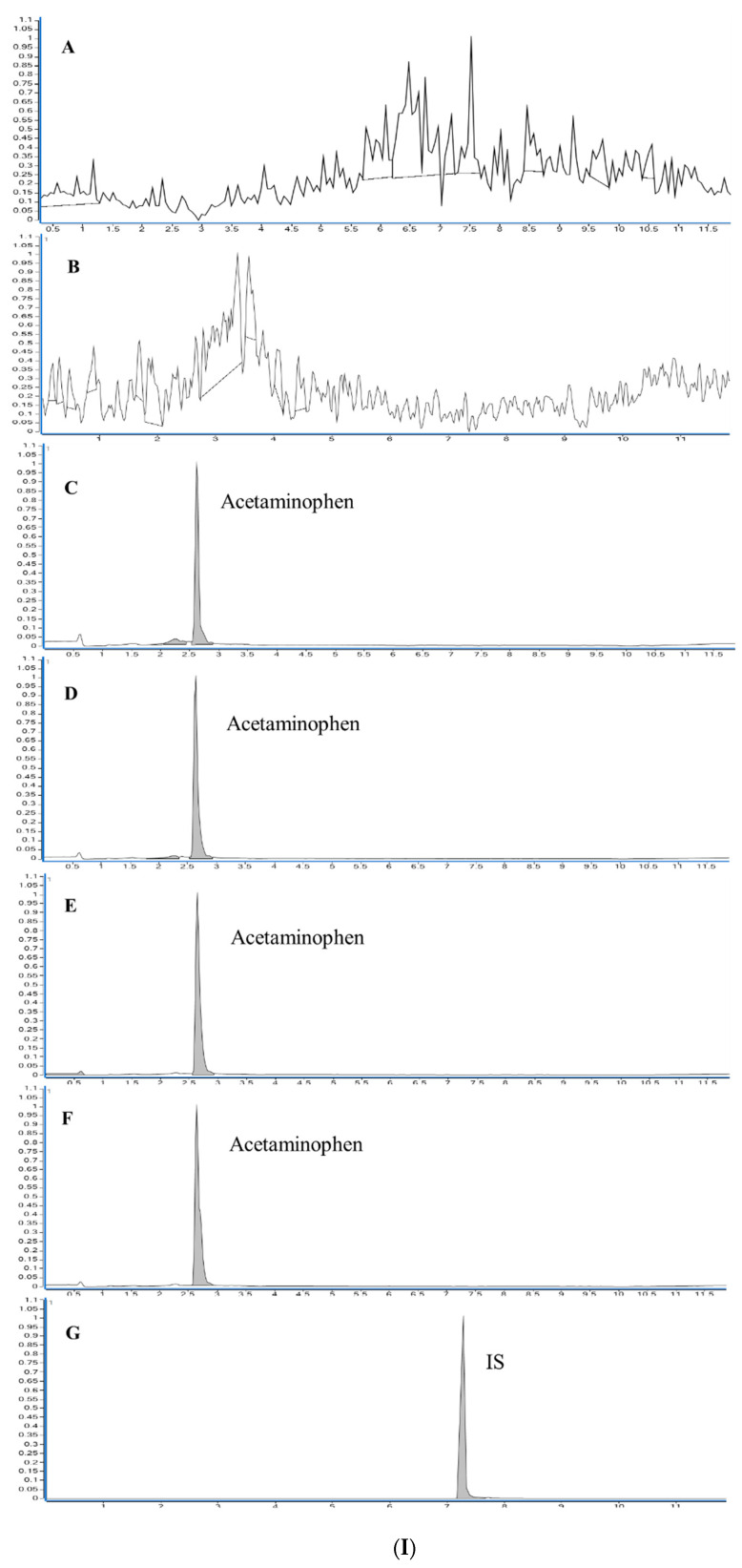

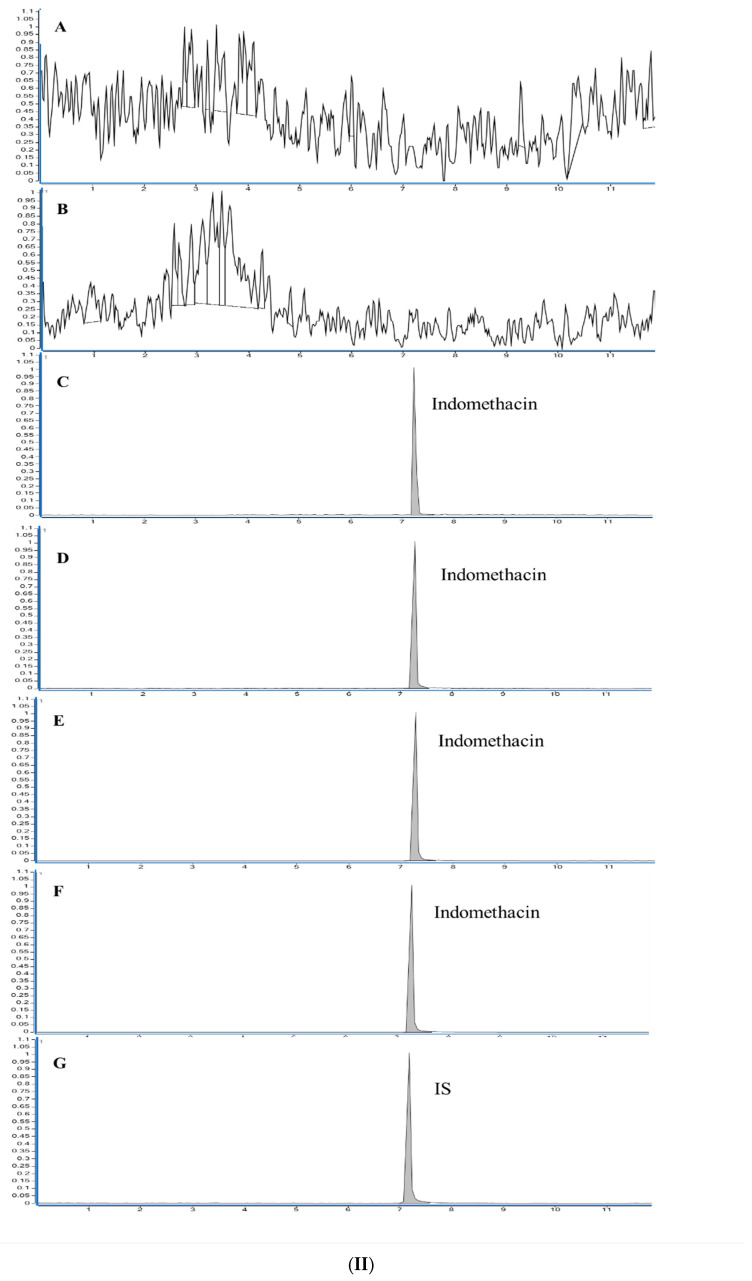
(**I**) Selected ion chromatograms of (**A**) solvent blank (**B**) plasma blank (**C**) LLOQ (1 ng/mL) (**D**) LQC (3 ng/mL) (**E**) MQC (300 ng/mL) (**F**) HQC (600 ng/mL) of acetaminophen (206 min) and (**G**) indomethacin (50 ng/mL) as I.S. (7.2 min). (**II**) Selected ion chromatograms of (**A**) solvent blank (**B**) plasma blank (**C**) LLOQ (1 ng/mL) (**D**) LQC (3 ng/mL) (**E**) MQC (250 ng/mL) (**F**) HQC (400 ng/mL) of Indomethacin (7.2 min) and (**G**) diclofenac sodium (50 ng/mL) as I.S. (7.1 min).

Indomethacin and acetaminophen were extracted by 3D-printed sorbent from rat plasma and analyzed quantified using a validated LC-MS/MS method to determine their pharmacokinetic profiles. There was no significant interference at the retention times of indomethacin, acetaminophen, and the I.S. in the chromatograms. The chromatograms of blank plasma, blank plasma spiked with indomethacin, acetaminophen, and diclofenac are shown in Figure 8(I,II). The retention times for indomethacin, acetaminophen, and diclofenac were 7.2 min, 2.6 min, and 7.1 min, respectively. The LC-ESI-MS/MS spectrum of the plasma spiked with indomethacin, acetaminophen, and diclofenac showed [M + H]^+^ ions at *m/z* 358, [M + H]^+^ ions *m/z* 152, and [M + H]^+^ ions at *m*/*z* 296. The samples were observed using the subsequent Q1/Q3 transitions: m/z 358 > 110.9 for indomethacin, *m*/*z* 152 > 110 for acetaminophen, and *m/z* 296 > 215 for diclofenac sodium. Therefore, an LC-ESI-MS/MS method was developed and validated for the quantification of indomethacin and acetaminophen. Linear regression with a weighting factor (1/x) was used to establish a calibration curve for acetaminophen, and linear regression was used for indomethacin. Based on the calibration curve (y = 4.529123 × x + 0.332651 for indomethacin and y = 0.601740 × x + 0.028456 for acetaminophen), the peak area ratios between the drugs and the I.S. were linearly related to indomethacin and acetaminophen concentrations, with determination coefficients of 0.9945 and 0.9966, respectively. The LLOQ of indomethacin and acetaminophen was 1 ng/mL. The intra-and inter-day precisions for indomethacin ranged from 1.80% to 6.33% and 1.23% to 6.33%, respectively, whereas for acetaminophen ranged from 2.18% to 14.48% and 1.67% to 10.26%, respectively. The intra-and inter-day accuracies for indomethacin ranged from 93% to 98.33% and 93% to 98.95%, respectively, and for acetaminophen, from 85% to 104% and 89.40% to 98.57%, respectively. The intra-and inter-day accuracy (% recovery) and precision (%CV) of indomethacin and acetaminophen were within the acceptable limits, as shown in Table 1. The extraction recovery of indomethacin using the 3D-printed sorbent was 85.37 (±2.4), 81.35 (±1.05), and 80.93 (±3.2) at the LQC, MQC, and HQC concentrations, respectively. In comparison, the extraction recovery of acetaminophen at LQC, MQC, and HQC concentrations was found to be 84.81 (±1.83), 82.8 (±3.6), and 83.12 (±3.8), respectively (Table 2). The matrix factor was within the limit for indomethacin, and that for acetaminophen was within the limit. The accuracy percentages of six duplicate measurements of indomethacin and acetaminophen, following two- and four-fold dilutions, were found to be within an acceptable range of 85–115% of their intended concentrations. No carry-over effects were detected during the retention times of indomethacin, acetaminophen, and I.S. The mean percentage changes in plasma for indomethacin and acetaminophen during bench-top stability, post-operative, and during three freeze-thaw cycles were within acceptable limits (Table 3). 

**Figure 9:**
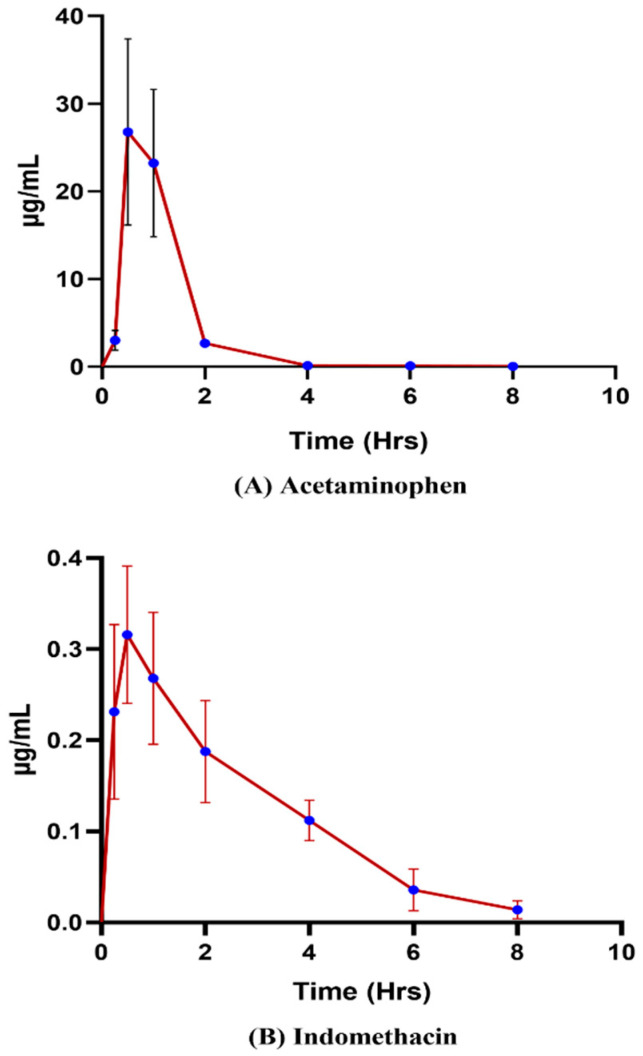
Pharmacokinetic profiles of (**A**) acetaminophen and (**B**) indomethacin.

An investigation into the pharmacokinetics of indomethacin and acetaminophen in rats was conducted through oral administration, employing LC-ESI-MS/MS analysis. Figure 9 illustrates the resulting pharmacokinetic profile, while Table 4 presents the computed pharmacokinetic parameters of both substances, obtained via PK Analysis Add-ins in the Microsoft Excel Program. The maximum drug concentration (C*_max_*) of indomethacin and acetaminophen was found to be 0.33 ± 0.04 µg/mL and 27.27 ± 9.9 µg/mL, respectively. The mean area under the curve (AUC_0–*t*_) for indomethacin is 0.93 µg h/mL and for acetaminophen is 32.33 µg h/mL. The time (T*_max_*) it takes for indomethacin and acetaminophen to reach the maximum concentration was found to be 0.5–1 h for both. Based on the obtained results, the developed 3D-printed sorbent efficiently extracted analytes from plasma, reduced sample preparation time, and minimized the use of organic solvents during sample preparation.

## 4. Conclusions

During the beginning of the pandemic, NSAIDs were commonly administered to COVID-19 patients in both hospital and non-hospital settings for alleviating symptoms such as fever, body aches, and headaches. As a result, certain NSAIDs have been deemed essential medicines by the WHO. It is essential to employ precise sample extraction techniques to accurately detect and quantify trace levels of NSAIDs in biological fluids because of the serious safety hazards associated with their use. Integrating cutting-edge technologies with 3D printing has revolutionized the development of advanced analytical sample preparation methods. This combination of technologies allows for the creation of sophisticated sample preparation techniques for the bioanalysis of compounds that are more efficient and cost-effective than traditional methods. In this study, we manufactured a customized filament through Hot Melt Extrusion (HME) by combining PVA, Affinisol^TM^ (HPMC HME 15 LV), and a TEC plasticizer. This indigenous filament was then employed as a 3D-printed sorbent to extract NSAIDs indomethacin and acetaminophen to determine the pharmacokinetic profile using LC-MS/MS. Furthermore, the use of 3D printing for sample preparation can provide new opportunities for research in prototyping next-generation solid-phase extraction cartridges or biological sample analysis. With the advancement of 3D printing and its utilization in sample preparation, this technology will undoubtedly continue to revolutionize the preparation of samples for analysis.

## Figures and Tables

**Table 1:** Determination (*n* = 6) of Intra-and inter-day precision and accuracy.

		Intra-Day Precision and Accuracy				Inter-Day Precision and Accuracy			
	QC Sample	LLOQ	LQC	MQC	HQC	LLOQ	LQC	MQC	HQC
	Theoretical Concentration (ng/mL)	1	3	300	600	1	3	300	600
Acetaminophen	Mean estimated concentration (ng/mL) ± SD	1.04 ± 0.06	2.55 ± 0.37	293.55 ± 7.87	586.02 ± 12.75	0.98 ± 0.05	18.23 ± 0.82	741.46 ± 59.43	1260.97 ± 57.12
	Precision (CV, %)	5.93	14.48	2.68	2.18	5.04	10.26	2.95	1.67
	Accuracy (%) ± SD	104 ± 6.16	85 ± 12.30	97.85 ± 2.62	97.67 ± 2.13	97.67 ± 4.92	94.33 ± 9.68	89.40 ± 2.64	98.57 ± 1.65
Indomethacin	QC Sample	LLOQ	LQC	MQC	HQC	LLOQ	LQC	MQC	HQC
	Theoretical concentration (ng/mL)	1	3	250	400	1	3	250	400
	Mean estimated concentration (ng/mL) ± SD	0.98 ± 0.04	2.95 ± 0.09	242.92 ± 4.38	384.94 ± 11.34	0.93 ± 0.06	2.79 ± 0.11	247.37 ± 4.62	377.05 ± 5.85
	Precision (CV, %)	6.33	3.08	1.80	2.34	6.33	3.86	1.87	1.23
	Accuracy (%) ± SD	93 ± 5.89	98.33 ± 3.03	97.17 ± 1.75	96.99 ± 2.27	93 ± 5.89	93.11 ± 3.59	98.95 ± 1.85	95.41 ± 1.17

**Table 2:** Determination (*n* = 6) of extraction recovery of analytes present in plasma.

Acetaminophen		Recovery		
	QC Sample	LQC	MQC	HQC
	Theoretical concentration (ng/mL)	3	300	600
	Mean % recovery ± SD	84.81 ± 1.83	82.8 ± 3.6	83.12 ± 3.8
	% CV	2.16	4.34	4.67
Indomethacin	QC Sample	LQC	MQC	HQC
	Theoretical concentration (ng/mL)	3	250	400
	Mean % recovery ± SD	85.37 ± 2.4	81.35 ± 1.05	80.93 ± 3.2
	% CV	2.9	1.3	4.05

**Table 3:** Stability data of indomethacin and acetaminophen in rat plasma (*n* = 3).

		Indomethacin			Acetaminophen		
	QC Sample	Theoretical Concentration (ng/mL)	Mean Estimated Concentration (ng/mL)± SD	Precision (CV, %)	Theoretical Concentration (ng/mL)	Mean Estimated Concentration (ng/mL)± SD	Precision (CV,%)
Bench-top stability	LQC	3	2.98 ± 0.11	3.64	3	2.91 ± 0.16	5.35
	HQC	400	401.61 ± 7.80	1.94	600	592.92 ± 10.80	3.60
Autosampler stability	LQC	3	2.99 ± 0.04	1.44	3	2.92 ± 0.15	5.20
	HQC	400	391.82 ± 6.72	1.72	600	596.26 ± 8.41	1.41
Three freeze-thaw cycles (each at −20 °C)	LQC	3	2.96 ± 0.12	4.01	3	3.01 ± 0.09	3.02
	HQC	400	395.72 ± 13.06	3.30	600	591.92 ± 6.66	1.13

**Table 4:** Pharmacokinetic parameters to assess the extraction efficiency of 3D sorbent using acetaminophen and indomethacin.

PK Parameters	Acetaminophen	Indomethacin
	Mean± SD	Mean± SD
*C*_max_ (µg/mL)	27.27± 9.9	0.33 ± 0.04
*t*_max_ (h)	0.5–1	0.5–1
*t_1/2_* (h)	0.81 ± 0.1	1.79 ± 0.44
AUC_0–t_ (min µg/mL)	32.33 ± 10.8	0.93 ± 0.17
AUC_0–ꝏ_ (min µg/mL)	32.38 ± 10.8	0.97 ± 0.2
K_el_ (h^−1^)	0.85 ± 1.12	0.41 ± 0.11

## Data Availability

Data will be available on request.
